# Atlantoaxial instability in psoriatic arthritis: Frequency and correlated factors from a single-center cohort

**DOI:** 10.1007/s10067-026-08111-0

**Published:** 2026-04-13

**Authors:** Mete Pekdiker, Huban Yilmaz, Sertaç Ketenci, Mete Kara

**Affiliations:** 1 Faculty of Medicine, Department of Internal Medicine, Division of Rheumatology, Hatay Mustafa Kemal University, Hatay, Türkiye; 2 Faculty of Medicine, Department of Internal Medicine, Hatay Mustafa Kemal University, Hatay, Türkiye; 3 Faculty of Medicine, Department of Physical Therapy and Rehabilitation, Division of Rheumatology, Ondokuz Mayıs University, Samsun, Türkiye; 4 Department of Internal Medicine, Division of Rheumatology, Izmir Bozyaka Training and Research Hospital, Izmir, Türkiye

**Keywords:** Atlantoaxial joint, Cervical vertebra, Psoriatic arthritis, Radiography

## Abstract

**Objectives:**

There is very limited data regarding atlantoaxial instability (AAI) in patients with psoriatic arthritis (axPsA). In this study, we aimed to contribute to the existing literature on this topic.

**Methods:**

Adult patients were included in this single-center study who were classified as PsA by the ‘CASPAR’ criteria and evaluated as having axial involvement according to the ‘Calin’ criteria. Those with inflammatory or non-inflammatory diseases that could affect the spine were excluded. Electronic patient files were reviewed retrospectively. Lateral neutral/full extension/full flexion and open-mouth anteroposterior cervical radiographs were evaluated by three rheumatologists blinded to the patients. Patients were compared in two groups as AAI-positive and AAI-negative.

**Results:**

A total of 100 patients with a mean age of 48.8 years and a mean PsA duration of 7.4 years, 57% of whom were female, were included in the study. A total of 20 AAI lesions were detected in 18% patients; subaxial subluxation was detected in eight, anterior atlantoaxial subluxation (AAS) in seven, posterior AAS in three, lateral AAS in one, and vertical subluxation in one case. In the group with AAI, the presence of psoriasis (Ps) (p = 0.037), scalp psoriasis (p < 0.001), and the use of targeted therapy for Ps and PsA (p < 0.001, p < 0.001) were significantly higher than in the AAI-negative group.

**Conclusion:**

Given that Ps and PsA patients on targeted therapy may reflect cases with higher disease activity and inadequate response to conventional treatments, it may be appropriate to consider closer monitoring for AAI in these patients.

**Table:** 

**Key Points** • *Atlantoaxial instability is present in approximately one-fifth of patients with axial psoriatic arthritis*. • *The most common instability lesion is subaxial subluxation, accounting for 40% of all lesions*. • *The presence of psoriasis, scalp psoriasis, and the use of targeted therapies for psoriatic arthritis or psoriasis are significantly more frequent in the group with atlantoaxial instability. These factors may be useful for cervical spine monitoring in patients with axial psoriatic arthritis*. *• The use of targeted therapies for psoriatic arthritis or psoriasis may indirectly indicate an association between high disease activity and atlantoaxial instability*.

## Introduction

Psoriatic arthritis (PsA) is a chronic inflammatory disease with a heterogeneous clinical spectrum. As a rheumatologic disease in the spondyloarthritis (SpA) group, PsA affects 30% of patients with psoriasis (Ps). Its prevalence is between 0.10–0.25%, and it generally affects adult individuals between the ages of 30–60 and is seen equally in both sexes. Its etiopathogenesis is multifactorial, and environmental factors, especially genetic factors, are also effective. Different structures such as joints, entheses, tendons, skin, and nails can be target tissues in PsA. Psoriatic arthritis, which is associated with erosive arthropathy, is associated with functional disability, loss of workforce, and decreased quality of life, similar to rheumatoid arthritis (RA) [1].

Although PsA is generally characterized by peripheral joint involvement, axial involvement is seen in 5–28% of early PsA cases and 25–70% of established PsA cases. Axial PsA (axPsA) can be characterized by spinal inflammation accompanied by sacroiliitis similar to ankylosing spondylitis (AS), or it can proceed with spondylitis without sacroiliitis. Cervical involvement is seen in 35–75% of PsA cases; it is correlated with long disease duration and disease severity. While upper cervical vertebra involvement can lead to AAI with C1-C2 arthritis, involvement of the lower vertebrae is generally characterized by new bone formation (syndesmophyte) [2].

Atlantoaxial instability (AAI) is a complication that can be encountered in all inflammatory arthritis group diseases (RA, AS, PsA) [3]. It can present with different clinical pictures, from asymptomatic forms to neurological complications that can lead to death. It can occur in different radiological forms, including atlantoaxial subluxation (AAS: anterior (a), lateral (l), posterior (p)), subaxial subluxation (SAS), and vertical subluxation (VS) [4]. The pathogenesis of AAI in the inflammatory arthritis group of diseases is not clearly known, but it has been associated with factors such as C1-C2 synovitis, juxta-articular bone erosion, joint ankylosis, local osteoporosis, pannus formation, and ligamentous laxity [5, 6].

Psoriatic arthritis can affect the cervical spine both like AS and can lead to destructive spondylitis in the way that RA causes damage such as erosion/subluxation in peripheral joints [7]. In the current literature, there are not enough studies investigating the frequency and risk factors of AAI in PsA disease, which can frequently involve the cervical spine and cause erosive changes in this region. The data in the literature are generally in the form of case reports, and there is no study examining all forms of AAI [8, 9]. In addition, there is no consensus on when to monitor for AAI in PsA cases. Our aim in this study is to contribute to the management of PsA in terms of this serious complication by revealing the frequency of AAI and associated factors in axPsA cases.

## Materials and methods

### Patient selection and study design

Our single-center, cross-sectional, and retrospective study was conducted in 2024 with adult axPsA patients who presented to the rheumatology unit of our university hospital, a tertiary care center. Patients were classified as ‘PsA’ according to the CASPAR criteria [10]. Cases with axial PsA were evaluated as those who met the ‘CALIN’ criteria among PsA cases [11]. Patients with inflammatory rheumatic diseases that can involve the cervical spine (such as rheumatoid arthritis, enteropathic arthritis), non-inflammatory diseases that can involve the cervical spine (such as diffuse idiopathic skeletal hyperostosis, ochronosis), a history of trauma or surgery that disrupts the integrity of the cervical spine, and spinal limitation to the extent that they could not undergo functional cervical radiography were excluded from the study. Demographic, clinical, laboratory, and treatment data were noted from electronic patient files. A family history of Ps or PsA was noted among first- or second-degree relatives. Biologic disease-modifying anti-rheumatic drugs (bDMARD) and targeted synthetic disease-modifying anti-rheumatic drugs (Janus kinase inhibitors: tsDMARDs) were defined as ‘targeted therapy’.

### Assessments

Rheumatoid factor (RF) was measured by the nephelometric method, and results ≥ 14 IU/ml in serum samples were considered positive. Anti-cyclic citrullinated peptide antibody-IgG (anti-CCP) was measured by the ELISA method, and results ≥ 5 U/ml in serum samples were considered positive. HLA-B27 was tested from peripheral venous blood by the ‘Real-time PCR’ method.

The degree of sacroiliitis was evaluated with a Ferguson X-ray, defined according to the Modified New York Criteria [12], and the highest grade of sacroiliitis lesion was noted. Syndesmophyte formation was evaluated as the presence of new bone formation that bridges two adjacent vertebrae [13]. Osteoproliferative lesions, acro-osteolysis, pencil-in-cup deformity, fluffy periostitis, large erosions in the joints, and joint ankylosis in anteroposterior hand or foot radiographs were evaluated as ‘erosive arthritis’ findings of peripheral joint involvement of PsA [14]. Atlantoaxial instability lesions were evaluated by reading four different X-rays: lateral neutral/full extension/full flexion radiographs and an open-mouth anteroposterior cervical radiograph.

Cervical spine involvement was divided into 3 main subtypes:

- Atlantoaxial subluxation (AAS):
  I. Anterior AAS (aAAS): the distance between the anterior surface of the odontoid process of C2 and the posteroinferior aspect of the C1 tubercle (anterior atlanto-dental distance) being > 3 mm on a lateral cervical radiograph [15]
  II. Posterior AAS (pAAS): the posterior surface of the anterior arch of the atlas being located behind the anterior C2 vertebral line due to dens erosion on a lateral radiograph [16]
  III. Lateral AAS (lAAS): C1 shifting more than 2 mm over C2 on an open-mouth radiograph [17]
- Vertical subluxation (VS): Chamberlain's line (the distance between the hard palate and the posterior edge of the foramen magnum) being > 3 mm [18]
- Subaxial subluxation (SAS): horizontal displacement of one vertebra more than ≥ 3 mm towards an adjacent vertebra without osteophyte formation [19]

Radiographic examinations performed within the last year were evaluated by three rheumatologists (M.P., S.K., M.K.) blinded to the patients. Full agreement was sought among the readers for the radiographic result; in case of disagreement, the radiographs were re-evaluated and a final decision was made. Interobserver agreement for the presence of atlantoaxial instability was calculated using Fleiss’ kappa coefficient. Data on the diagnosis and clinical findings of Ps were obtained through examination by a dermatologist with over ten years of experience.

### Statistical analysis

Statistical analyses were performed using IBM SPSS Statistics for Windows, Version 27.0 (IBM Corp., Armonk, NY, USA). Descriptive statistics for continuous variables were reported as mean ± standard deviation (SD), and categorical variables were reported as number and percentage (%). The distribution characteristics of the data were examined with the Kolmogorov–Smirnov test, and histograms and Q-Q plots were also reviewed to support the appropriateness of the distribution. In normally distributed variables, intergroup comparisons were made with the independent samples t-test, while the Mann–Whitney U test was preferred for non-normally distributed variables. Differences between categorical variables were evaluated using the Pearson chi-square test or Fisher's exact test, depending on the expected cell values. A significance level of p < 0.05 was accepted in all analyses. The study was conducted in accordance with the principles of the Declaration of Helsinki and was approved by the Non-interventional Clinical Research Ethics Committee of the center where the study was conducted at its meeting on 02.19.2025 (Meeting Number: 03, Decision Number: 36).

## Results

A total of 129 cases diagnosed with axial PsA were screened from electronic patient files; five patients were excluded due to cervical spine limitation preventing functional cervical radiography, 12 patients due to lack of radiographic examinations, 10 patients due to lack of laboratory data, and two patients due to concomitant diffuse idiopathic skeletal hyperostosis. A total of 100 patients with a mean age of 48.8 ± 10.3 years were included in the study. Of the study group, 43% were male and 57% were female, and 28% had a history of smoking. The frequency of comorbidities was 31% for hypertension, 17% for diabetes mellitus, and 16% for coronary artery disease.

The mean PsA duration of our study group was 7.4 ± 6.9 years, and the mean age of first symptom onset was 41.5 ± 11.7 years. While 67% of the cases had peripheral joint involvement, 37% had erosive arthritis in the peripheral joints. HLA-B27, RF, and anti-CCP positivity were 25%, 9%, and 13%, respectively. In a total of nine cases with rheumatoid factor positivity, anti-CCP positivity was concomitant in five cases, and erosive arthritis was present in six (66%) of them, and in seven (54%) of the 13 cases with anti-CCP positivity. In the radiographic evaluation of the sacroiliac joint, the rates of patients with Stage 0/1/2/3/4 sacroiliitis were 33%, 18%, 24%, 21%, and 4%, respectively; and 36% of the cases had syndesmophyte formation.

Psoriasis was present in a total of 82 patients, and the diagnosis of Ps was confirmed by skin biopsy in 77 patients. Family history of Ps/PsA was present in 31% of patients. Regarding the timing of skin-joint involvement, 69% of the cases had Ps first, 10% had simultaneous onset, and 21% had articular-onset. The rate of patients using targeted therapy for PsA was 54%, with secukinumab being the first in line (18%). In 56% of the cases, there was a history of bDMARD use for the treatment of Ps. Table 1 presents the demographic, clinical, laboratory, and treatment data of the study group.

**Table 1:** Demographic, clinical, laboratory, and treatment characteristics of the study population

Total patients, n	100
Age, years, mean ± SD	48.8 ± 10.3
Male sex, n (%)	43 (43)
Female sex, n (%)	57 (57)
Smoking history, n (%)	28 (28)
Hypertension, n (%)	31 (31)
Diabetes mellitus, n (%)	17 (17)
Coronary artery disease, n (%)	16 (16)
Family history for Ps or PsA, n (%)	31 (31)
PsA duration, years, mean ± SD	7.4 ± 6.9
Age at first symptom of arthritis, years, mean ± SD	41.5 ± 11.7
Peripheral arthritis, n (%)	67 (67)
Erosive arthritis, n (%)	37 (37)
HLA-B27 positivity, n (%)	25 (25)
RF positivity, n (%)	9 (9)
Anti-CCP positivity, n (%)	13 (13)
Grade of sacroiliitis, n (%)	
-Grade 0	33 (33)
-Grade 1	18 (18)
-Grade 2	24 (24)
-Grade 3	21(21)
-Grade 4	4 (4)
Patients with syndesmophyte, n (%)	36 (36)
Timing of skin-joint involvement	
-Psoriasis-onset	69 (69)
-Simultaneous onset	10 (10)
-Articular-onset	21 (21)
Psoriasis, n (%)	82 (82)
Psoriasis duration, years, mean ± SD	13.7 ± 9.7
Nail psoriasis, n (%)	15 (15)
Scalp psoriasis, n (%)	29 (29)
Targeted therapy for psoriasis, n (%)	56 (56)
Targeted therapy for PsA, n (%)	54 (54)
-Etanercept, n (%)	1 (1)
-Adalimumab, n (%)	6 (6)
-Infliximab, n (%)	1 (1)
-Certolizumab pegol, n (%)	6 (6)
-Golimumab, n (%)	2 (2)
-Secukinumab, n (%)	18 (18)
-Ixekizumab, n (%)	8 (8)
-Tofacitinib, n (%)	5 (5)
-Upadacitinib, n (%)	3 (3)
-Risankizumab, n (%)	3(3)
-Bimekizumab, n (%)	1(1)

Abbreviations: SD, standard deviation; PsA, psoriatic arthritis; Ps, psoriasis; RF, rheumatoid factor; Anti-CCP, anti-cyclic citrullinated peptide antibody-IgG

**Fig. 1:**
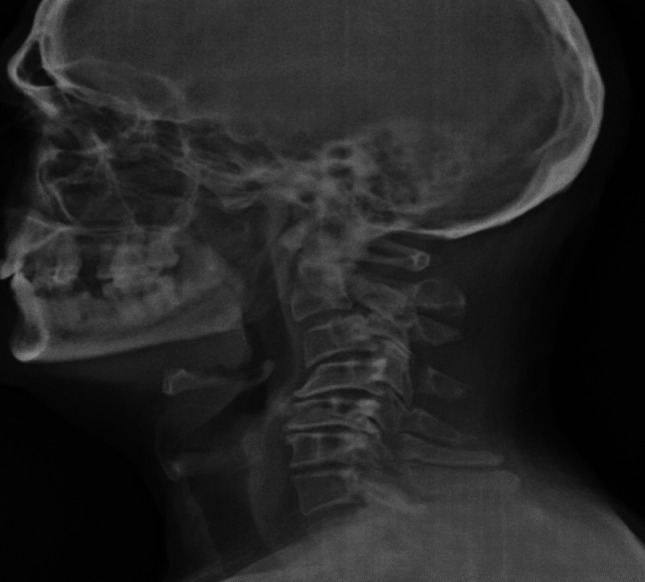
A 50-year-old male patient receiving biologic therapy for psoriasis, who was asymptomatic with respect to joint involvement, was incidentally found to have a subaxial subluxation lesion during evaluation at our clinic

 Eighteen percent of the patients in our study group had AAI. We detected mixed-type lesions as aAAS + pAAS in one case and aAAS + VS in another case. The most common AAI lesion we detected was SAS with eight cases, constituting 40% of all AAI lesions. The second most common AAI lesion was aAAS (n = 7). Posterior AAS was detected in three cases, lAAS in one case, and VS in one case. Interobserver agreement among the three rheumatologists was good, with a Fleiss’ kappa value of 0.78, indicating substantial agreement. Table 2 presents the AAI data of the study group. None of the cases with AAI had neurological symptoms or findings. Figure 1 shows the lateral neutral cervical radiograph of a fifty-year-old male case using biological DMARD for Ps; an example case who was asymptomatic in terms of joint findings and in whom SAS was incidentally detected in our clinic.

**Table 2:** Atlantoaxial instability data of study population

Patients with atlantoaxial instability, n (%)	18 (18)
Total number of atlantoaxial instability lesions, n (%)	20 (100)
-Anterior atlantoaxial subluxation	7 (35)
-Posterior atlantoaxial subluxation	3 (15)
-Lateral atlantoaxial subluxation	1 (5)
-Subaxial subluxation	8 (40)
-Vertical subluxation	1 (5)

When the two groups with and without AAI were compared, the presence of Ps (p = 0.037), scalp psoriasis (p < 0.001), a history of using targeted therapy for PsA (p < 0.001), and a history of using targeted therapy for Ps (p < 0.001) were significantly higher in the AAI-positive group than in the AAI-negative group. Laboratory parameters such as age, gender, smoking, family history, duration of PsA or Ps, peripheral arthritis, erosive arthritis, HLA-B27/RF/anti-CCP, sacroiliitis stage, and the presence of syndesmophytes were similar in both groups (Table 3).

**Table 3:** Comparison of the study population according to atlantoaxial instability status

Variables		AAI-positive group (n = 18)	AAI-negative group (n = 82)	P-value
Age, years, mean ± SD		48.7 ± 11.9	48.9 ± 10	0.923 ^t^
Male sex, n (%)		7 (38.9)	36 (43.9)	0.697 ^χ2^
Smoking history, n (%)		5 (27.8)	23 (28)	0.981 ^χ2^
Family history for Ps or PsA, n (%)		4 (22.2)	27 (32.9)	0.374^χ2^
PsA duration, years, mean ± SD		8.1 ± 5	7.3 ± 7.3	0.679 ^t^
Age at first symptom of PsA, years, n (%)		40.6 ± 13.6	41.7 ± 11.3	0.721^t^
Psoriasis, n (%)		18 (100)	64 (78)	**0.037**^**f**^
Psoriasis duration, years, mean ± SD		13.2 ± 8.2	13.8 ± 10.1	0.223^m^
Nail psoriasis, n (%)		3 (16.7)	12 (18.8)	> 0.999^f^
Scalp psoriasis, n (%)		18 (100)	11 (17.2)	** < 0.001**^**χ2**^
Peripheral arthritis, n (%)		13 (72.2)	54 (65.9)	0.603^χ2^
Erosive arthritis, n (%)		10 (55.6)	27 (32.9)	0.072^χ2^
HLA-B27 positivity, n (%)		5 (27.8)	20 (24.4)	0.769 ^f^
RF positivity, n (%)		0 (0)	9 (11)	0.357 ^f^
Anti-CCP positivity, n (%)		2 (11.1)	11 (13.4)	0.574 ^f^
Targeted therapy for PsA, n (%)		18 (100)	36 (43.9)	** < 0.001 **^f^
Targeted therapy for Ps, n (%)		18 (100)	38 (59.4)	** < 0.001 **^f^
Patients with syndesmophyte, n (%)		8 (44.4)	28 (34.1)	0.410 ^f^
Grade of sacroiliitis, n (%)				
	-Grade 0	5 (27.8)	28 (34.1)	0.792^χ2^
	-Grade 1	3 (16.7)	15 (18.3)	
	-Grade 2	5 (27.8)	19 (23.2)	
	-Grade 3	5 (27.8)	16 (19.5)	
	-Grade 4	0 (0)	4 (4.9)	

Abbreviations: *AAI* atlantoaxial instability; *SD* standard deviation; *RF* rheumatoid factor; *Anti-CCP* anti-cyclic citrullinated peptide antibody-IgG; *PsA* psoriatic arthritis; *Ps* psoriasis

*t* independent samples t-test, *χ*^*2*^ Pearson’s chi-square test, *f* Fisher’s exact test, *m* Mann–Whitney U test,

A p value < 0.05 was considered statistically significant

## Discussion

In this study, we investigated the frequency of AAI, a critical lesion of the cervical spine, which is an important target region for axPsA, and its associated factors. In our study, which has the largest number of patients among the studies conducted for this purpose to date, we found the frequency of AAI to be 18% in axPsA cases. The most common AAI lesion was SAS, which constituted 40% of all AAI lesions. The presence of Ps, scalp psoriasis, and the use of targeted therapy for Ps and PsA were significantly higher in AAI-positive axPsA cases compared to the AAI-negative group. Considering that b/tsDMARDs are generally prescribed for patients with inadequate response to conventional synthetic DMARDs (csDMARDs) based on the decision of a rheumatologist or dermatologist, this finding may indirectly suggest an association between AAI and higher disease severity in joint and/or skin involvement in patients with PsA. The fact that there is currently no consensus on the measurement of axPsA activity and no monitoring recommendation for AAI makes these results even more meaningful.

Blau RH et al. divided the radiographic cervical spine involvement in PsA cases into two groups as AS-like ‘ankylosing form’ and RA-like ‘erosive form (apophyseal and odontoid erosion, axial and subaxial subluxation)’. In their study involving a total of 28 patients, they detected AAI in seven cases, three of which had SAS [20]. Lassoued S et al. found the rate of patients with aAAS to be 23% in their study including 56 PsA cases [21]. Salvarani C et al. found radiographic changes in the cervical spine in 70% (n = 40) of 57 patients with a mean age of 56 years, a mean arthritis duration of 116 months, and a mean Ps duration of 175 months; the most common lesions were narrowing in the subaxial spine (n = 35) and ossification in the anterior longitudinal ligament (n = 30). In this study, AAS was detected in 13 patients and SAS in 16 patients, and similar to our study, SAS was the most common AAI lesion [7]. Jenkinson T et al. found cervical spine involvement in 45% of 75 PsA cases radiographically. In the evaluation for AAI lesions, they detected AAS in three cases and SAS in one case. The researchers defined the duration of psoriatic arthropathy as the most important determinant of cervical spine disease but did not find it to be associated with the severity of PsA, or the extent of skin or nail involvement [22]. Jeannou J et al. investigated the radiographic cervical involvement of 30 PsA cases with inflammatory axial pain with a mean disease duration of 80 months and detected aAAS in 10% (n = 3) of the cases [23]. The previous five studies did not include data on risk factors for AAI lesions or AAS subtypes; the frequencies of aAAS and SAS in our study group (7% and 8%, respectively) were in between the values of these studies [7, 20–23].

In a study by Bobek D et al. where they radiographically investigated cervical spine changes in 41 PsA patients, the most common lesion was apophyseal joint changes, and SAS was seen in only one case [24]. In the study by Laiho K et al., in which AAI lesions in PsA cases were evaluated in a wide radiographic spectrum, aAAS was detected in five cases (8%), VS in three cases (5%), SAS in two cases (3%), and aAAS + VS in two cases (3%) in 65 PsA cases with a mean age of 49 years, while no pAAS was detected in any case [25]. Laiho K et al. examined the subtypes of AAI in detail and reported a frequency of 19%, which is comparable to our findings. In a study where craniocervical junction involvement was examined by radiography, CT, or MRI in patients with inflammatory arthritis, it was found that craniocervical junction involvement was higher in RA (49.5%) and SpA (26.5%) cases compared to PsA (18.2%) (RA vs PsA: p < 0.001, SpA vs PsA: p < 0.001). In this study, odontoid process involvement was detected in two cases, AAS in two cases, SAS in 6 cases, and VS in one case in a total of 55 PsA patients [3].

The study most similar to ours in terms of patient selection and number was conducted by Queiro R et al. They observed changes in the cervical spine in 41% of 100 PsA cases with radiographic sacroiliitis and saw arthritis duration and peripheral erosions more frequently in the group with cervical spine involvement. In our study, no relationship was found between these two variables and AAI, but in both studies, there is no relationship between gender, HLA-B27, peripheral joint involvement, and the degree of sacroiliitis and cervical involvement. In the study by Queiro R et al., syndesmophytes were detected in 19% (n = 8), SAS in 24% (n = 10), and AAS in 2.4% (n = 1) of the cases, while the most common lesion was facet joint erosion/fusion with 34% (n = 14); the frequency of AAI was found to be lower than in our study (18% vs 11%) [26].

In the only prospective study, AAS was detected in 2.2% of 1534 PsA cases, and in the AAS-positive group, PASI (Psoriasis Area Severity Index), Basal Modified Steinbrocker Score, number of radiographically damaged joints, sacroiliitis, and erythrocyte sedimentation rate elevation were statistically higher. The greater need for targeted therapy for Ps and PsA in our study shows an indirect similarity with this prospective cohort [27]. Factors such as bias in patient selection, use of different radiological criteria, different disease durations, and different definitions for cervical involvement are likely to lead to different results in studies investigating the frequency of AAI in axPsA patients in the literature. Lateral AAS is a rare condition in axPsA cases and has been reported in the form of case reports [28]; in our study, lAAS was detected in only one case. Another AAI lesion, pAAS, is a lesion that has been little studied in PsA cases in the literature and was reviewed in only one of the above studies [25]; in our study group, pAAS was present in three cases.

HLA-B27 is a genetic marker that is found in 90% of AS cases and is associated with SpA group diseases. While its frequency is 5% in psoriasis cases, it is 30–60% in PsA cases and is associated with a short time between Ps and joint involvement, early age of disease onset, axial disease, uveitis, dactylitis, male gender predominance, and poor prognosis [29]. In our study group, HLA-B27 positivity was 25% and was not associated with AAI. According to a meta-analysis, the frequency of anti-CCP antibodies in PsA cases is 10% and is associated with polyarthritis, joint erosion, and dactylitis [30]. The frequency of anti-CCP in our study population was 13% (n = 13), and 11 cases had polyarticular disease, seven cases had joint erosion, but there was no association with AAI. Rheumatoid factor positivity is 10% in PsA and is associated with an earlier age of onset, more entheseal damage, and higher BASDAI and DAPSA scores, but there is no data for axPsA [31]. The frequency of RF in our study population was 9% (n = 9), and all of the cases had polyarticular disease and six of them had erosive disease, but we did not find any relationship with AAI. Rheumatoid arthritis was excluded based on the 2010 ACR/EULAR classification criteria [32]. Although five patients with rheumatoid factor or anti-CCP positivity fulfilled these criteria, they were classified as psoriatic arthritis because they had clinically evident psoriasis and hand radiographs showing features not typical of rheumatoid arthritis but compatible with psoriatic arthritis, including distal interphalangeal joint involvement and a ‘mouse-ear’ appearance.

Psoriasis severity is correlated with both PsA development and PsA activity [33, 34], and in our study, the use of targeted synthetic DMARDs (i.e., the number of cases with high disease activity Ps) was significantly higher in the AAI-positive group. A family history of psoriasis has no effect on PsA disease activity [35], and in parallel with this, we did not find a relationship between the development of AAI and a family history of Ps in our study. The frequency of ‘PsA Sine Psoriasis’ in our study group was 18%, and in a multicenter study from Turkey, the frequency of ‘PsA Sine Psoriasis’ was found to be 16%, similar to our cohort [36]. Again, although scalp involvement is a predictive factor for the development of PsA, it is not a risk factor for PsA severity [37], and we did not find a relationship between scalp involvement and AAI. There is a relationship between nail involvement and distal interphalangeal joint arthritis in patients with PsA, and anatomical proximity forms the basis of this relationship [38]; the relationship between scalp involvement and AAI in our study may also be related to this anatomical theory. Half of the 18 patients in whom we detected an AAI lesion were asymptomatic, while the other half had only neck pain, and no patient with AAI had neurological symptoms or findings. This is an expected situation, and neurological symptoms/findings are rarely seen in AAI lesions in PsA patients [39].

Despite the results we obtained with detailed analyses in our study, the single-center retrospective design is an important limitation. Due to the relatively small number of AAI-positive cases (n = 18), multivariable regression analysis could not be reliably performed, as recommended events-per-variable thresholds for logistic regression were not met. Inter-observer and intra-observer reliability, the fact that structural changes/damage in the axial skeleton were not evaluated with a scoring system such as PASRI (Psoriatic Arthritis Spondylitis Radiological Index) [40], and the low sensitivity of conventional radiography, the imaging method used to detect AAI lesions, are important limitations of our study. The sensitivity of conventional radiography in detecting AAI lesions is lower than that of computed tomography and magnetic resonance imaging [41], and patients with cervical spine limitation that precluded the performance of functional radiography were also excluded. Therefore, the frequency of AAI may have been underestimated. The fact that the center where the study was conducted is a tertiary rheumatology unit creates an unintentional bias in patient selection.

In this study, in which all forms of AAI were investigated for the first time in axPsA cases in the literature, we found the presence of Ps, scalp psoriasis, and a history of b/tsDMARD use for Ps and PsA to be significantly higher in the AAI-positive group. Current rheumatology and dermatology treatment guidelines recommend b/tsDMARD treatment in cases resistant to conventional therapies [42, 43]. Therefore, patients with Ps and PsA with higher disease activity may potentially represent a group at increased risk for AAI. An AAI lesion was present in 18% of our study population with a long disease duration, and the most common one was SAS (8%). Therefore, it is not sufficient to evaluate only the C1-C2 joint relationship in axPsA patients. Prospective studies to be conducted with sensitive imaging methods are needed to better understand this serious involvement of PsA.

## Data Availability

The data underlying this article will be shared on reasonable request to the corresponding author.
